# Heterogeneous Distribution of Proton Motive Force in Nonheritable Antibiotic Resistance

**DOI:** 10.1128/mbio.02384-22

**Published:** 2023-01-04

**Authors:** Annie H. Lee, Rachit Gupta, Hong Nhi Nguyen, Isabella R. Schmitz, Deborah A. Siegele, Pushkar P. Lele

**Affiliations:** a Artie McFerrin Department of Chemical Engineering, Texas A&M University, College Station, Texas, USA; b Department of Chemical Engineering, University of Michigan, Ann Arbor, Michigan, USA; c Department of Biology, Texas A&M University, College Station, Texas, USA; California Institute of Technology

**Keywords:** persistence, flagellar motility, antibiotic resistance, efflux pumps, beta-lactams

## Abstract

Bacterial infections that are difficult to eradicate are often treated by sequentially exposing the bacteria to different antibiotics. Although effective, this approach can give rise to epigenetic or other phenomena that may help some cells adapt to and tolerate the antibiotics. Characteristics of such adapted cells are dormancy and low energy levels, which promote survival without lending long-term genetic resistance against antibiotics. In this work, we quantified motility in cells of Escherichia coli that adapted and survived sequential exposure to lethal doses of antibiotics. In populations that adapted to transcriptional inhibition by rifampicin, we observed that ~1 of 3 cells continued swimming for several hours in the presence of lethal concentrations of ampicillin. As motility is powered by proton motive force (PMF), our results suggested that many adapted cells retained a high PMF. Single-cell growth assays revealed that the high-PMF cells resuscitated and divided upon the removal of ampicillin, just as the low-PMF cells did, a behavior reminiscent of persister cells. Our results are consistent with the notion that cells in a clonal population may employ multiple different mechanisms to adapt to antibiotic stresses. Variable PMF is likely a feature of a bet-hedging strategy: a fraction of the adapted cell population lies dormant while the other fraction retains high PMF to be able to swim out of the deleterious environment.

## INTRODUCTION

Each year, approximately 24.6 million pounds of antimicrobials are used in raising food animals for human consumption, and 3.0 million pounds are used for human medication (1). Overuse of antibiotics often promotes antimicrobial resistance and has resulted in the rise of superbugs that can resist a variety of drugs. It is estimated that antibiotic-resistant pathogens caused ~5 million deaths in 2019 (2). When treating bacterial pathogens that are difficult to eradicate, clinicians may sequentially combine multiple antibiotics during treatment (3). Sequential therapy is considered to be a potent treatment approach for curing infections, as the fluctuating antibiotic stresses make it challenging for bacteria to adapt and survive (4–8). Nonetheless, increased cross-tolerance has been reported for sequential therapies: in many instances, exposure to a stressor, such as an antibiotic, appears to promote adaptation against subsequent stressors (9–12).

Although genetic inheritance of resistance is common, pathogens may also adapt and survive in the presence of antibiotics without acquiring resistant genes. The underlying causes may involve epigenetic phenomena that are transient. Examples of such nongenetic or nonheritable resistance include persister cells (13, 14), which are found in all phases of cell growth, making up ~0.0001 to 0.001% of the population in the exponential phase and ~1% in bacterial biofilms and stationary-phase cultures (15). Arrested growth in the presence of the antibiotic can be a characteristic of nonheritable resistance, a term that we use interchangeably with adaptive resistance or tolerance in this work. Once the antibiotic stressor is removed, the survivors may resume rapid growth to give rise to a new population that is genetically nondistinct from the original population (16). Sequential exposure to antibiotics increases the probability of adaptive resistance (17, 18). Despite it being linked to persistent infections, the current understanding of the different mechanisms of adaptive resistance is incomplete.

One mechanism by which cells can survive antibiotic stresses without acquiring resistance genes involves efflux pumps. These pumps utilize proton motive force (PMF) to expel antibiotics and decrease their intracellular levels, thereby protecting the cell (19, 20). PMF is an electrochemical gradient of protons across the cell membrane that powers major functions, including bacterial motility, cell division, and ATP synthesis (21). Interestingly, PMF also powers the uptake of certain antibiotics, making cells more susceptible to them. Cells with low PMF are often protected against aminoglycoside antibiotics by reductions in the uptake and effectiveness of antibiotics (22, 23). Dissipation of PMF using the protonophore carbonyl cyanide *m*-chlorophenyl hydrazine (CCCP) reportedly increases the occurrence of persister cells (17, 24). A decrease in ATP levels, which occurs when PMF is dissipated, has also been suggested to promote adaptive or nonheritable resistance (25). Thus, conditions that promote low PMF favor cell adaptation to antibiotic stresses.

In this work, we measured the phenotypic properties of cells of Escherichia coli that developed adaptive resistance following sequential exposure to two antibiotics, rifampicin and ampicillin. We chose this combination as it promotes nonheritable antibiotic tolerance (17), which enabled us to quantify motility specifically in adapted cells. The swimming speeds acted as quantitative probes of the PMF, as the latter powers the rotation of flagella, which enables motility (26–28). We observed that ~1 of 3 cells retained high swimming speeds following a brief exposure to rifampicin and then a sustained exposure to ampicillin for several hours. These motile cells were able to survive and subsequently grow once the antibiotic was removed. Our results indicate that cells with high and low PMF levels coexist in a subpopulation that has adapted to the antibiotic.

## RESULTS

### Motility is retained after sequential exposure to antibiotics.

**FIG 1:**
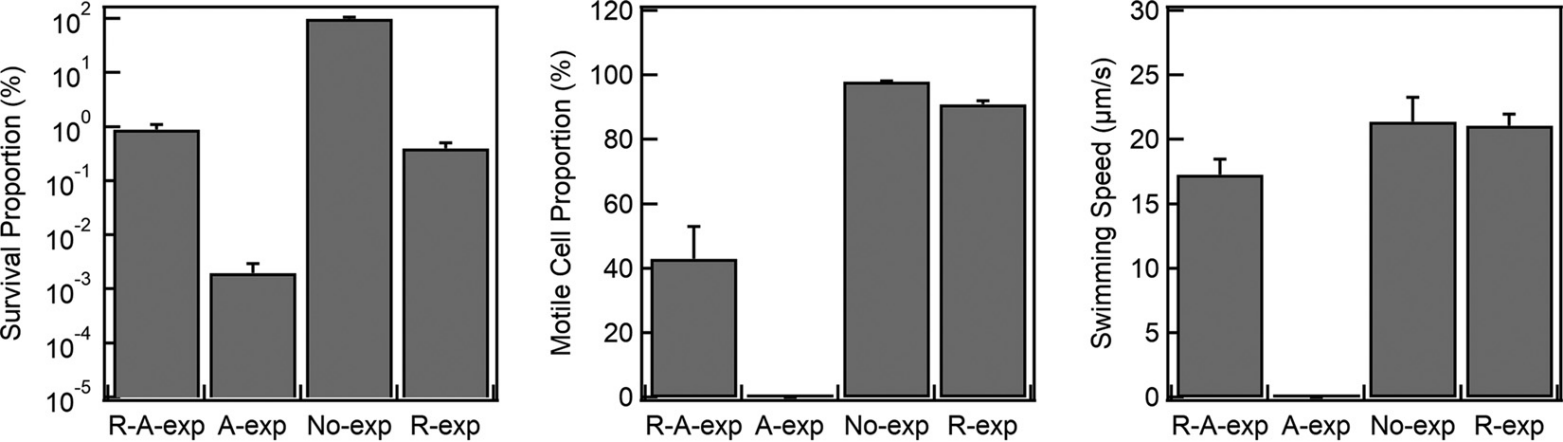
Surviving proportion (left), motile proportion (middle), and mean speed of swimming cells (right) for the sequential exposure to rifampicin-ampicillin (*R-A-exp*), the single exposure to ampicillin (*A-exp*), and the single exposure to rifampicin (*R-exp*). *No-exp* refers to untreated wild-type cells. The means and standard errors for the CFU assays were calculated over three or more independent biological replicates (three technical replicates per biological sample). The differences in the mean CFU/mL across all the groups were significant. Motile proportions were determined from ~80 to 350 cells per replicate, and swimming speeds were determined from ~30 to 160 cells per replicate (over three biological replicates). The mean speeds were not significantly different across the different treatments, except for *A-exp*, where no motile cells were observed.

We worked with wild-type E. coli strain RP437 as this strain has been widely employed for motility studies (29, 30). We sequentially exposed exponentially growing cells to two antibiotics: rifampicin and ampicillin. The working concentrations of antibiotics employed were several factors higher than the minimum inhibitory concentrations (MICs), as detailed in Materials and Methods. In the sequential treatment (*R-A-exp*), cells were first exposed for 30 min to rifampicin, which halts transcription by inhibiting RNA polymerase activity (31). We then replaced the rifampicin with ampicillin, a β-lactam that inhibits cell wall synthesis by binding to transpeptidase (32). This approach has been previously used by Kwan and coworkers to induce tolerance of antibiotics and enrich the survivors in culture (17). We performed another set of experiments in which exponentially growing cells were exposed to a single antibiotic, ampicillin (*A-exp*). The controls consisted of exponentially growing cells at an optical density at 600 nm (OD_600_) of 0.8 that were not exposed to any antibiotics (*No-exp*) and cells that were exposed to rifampicin only for 30 min (*R-exp*) (see Materials and Methods). After 3 h of exposure to ampicillin, the medium in which the cells were suspended was serially diluted with a phosphate-buffered saline (PBS) solution free of any antibiotics. The cells were then plated on lysogeny broth (LB) agar plates, and survival was quantified by counting the number of colony forming unit (CFU). In the *A-exp* treatment, only ~0.002% ± 0.001% (mean ± standard error) of the cells survived after the 3-h exposure to ampicillin relative to the survival in the *No-exp* control (Fig. 1). In comparison, ~0.9% ± 0.2% of the cells in the *R-A-exp* treatment survived after the 3-h exposure to ampicillin when compared to the survival of the *No-exp* cells. The majority of the cells perished during the short exposure to rifampicin, as confirmed by the *R-exp* control, where only ~0.4% ± 0.1% of the cells survived relative to the survival in the *No-exp* treatment. The higher survival fraction in the *R-A-exp* treatment relative to that in the *R-exp* treatment might be due to a weak antagonistic relationship between the two drugs. When the *R-A-exp* and the *A-exp* populations were plated on LB plates supplemented with ampicillin (100 μg/mL), no colonies grew, indicating that the cells did not develop permanent resistance to ampicillin.

Next, we performed motility assays by drawing samples from the *R-A-exp* and *A-exp* treatments without removing ampicillin. We quantified the fraction of swimming cells in the population and their speeds. As shown by the results in Fig. 1, the motile proportion in the *R-A-exp* cells was ~43% ± 10% after 3 h of exposure to ampicillin. In the motile population, ~55% of the cells swam freely, while ~45% of the cells appeared to adhere to the glass surface (33) and rotated in place as tethered cells do (34). As the rotation of the adhered body is powered by the flagella (33), we counted them as motile cells. No motile cells were observed in the *A-exp* population, whereas ~91% ± 1% of cells were motile in the *R-exp* population. In the *No-exp* control, ~98% ± 0.2% of cells were motile. Among the *No-exp* motile cells, ~94% of the cells swam freely; the remaining ~6% of the cells appeared tethered. Considering that the fractions of so-called tethered cells in the *No-exp* and *R-A-exp* cells were different, it is possible that the tethered cells consisted of those that sedimented and adhered because they lacked adequate flagellar power to sustain swimming or because they collided more often with the surface due to altered swimming characteristics, such as tumbling frequency (35, 36). Alternately, the cell surface may simply be more adherent in the *R-A-exp* population. As we could not distinguish between these possibilities, we focused on the swimming cells in all treatments and quantified the swimming speeds (Fig. 1): the mean speeds were 17.3 ± 1.2 μm/s (*R-A-exp*), 0.0 ± 0.0 μm/s (*A-exp*), 21.4 ± 1.9 μm/s (*No-exp*), and 21.1 ± 0.9 μm/s (*R-exp*). As motility is powered by PMF (26, 27), the relatively high swimming speeds of the *R-A-exp* cells suggested that the motile proportion consisted of cells with a high PMF. We were able to qualitatively reproduce the motility phenotype following *R-A-exp* treatment in two other strains of E. coli: strain AW405 and a motile isolate of strain BW25113 (SDB260) (see Movies S3 and S4 in the supplemental material).

10.1128/mbio.02384-22.8 MOVIE S3 *R-A-exp* cells of AW405 strain visualized after 3 h of ampicillin treatment. Download Movie S3, AVI file, 3.3 MB. Copyright © 2023 Lee et al. 2023 Lee et al. https://creativecommons.org/licenses/by/4.0/ This content is distributed under the terms of the Creative Commons Attribution 4.0 International license.

10.1128/mbio.02384-22.9 MOVIE S4 *R-A-exp* cells of SDB260 strain (BW25113 background) visualized after 3 h of ampicillin treatment. Download Movie S4, AVI file, 2.0 MB. Copyright © 2023 Lee et al. 2023 Lee et al. https://creativecommons.org/licenses/by/4.0/ This content is distributed under the terms of the Creative Commons Attribution 4.0 International license.

**FIG 2:**
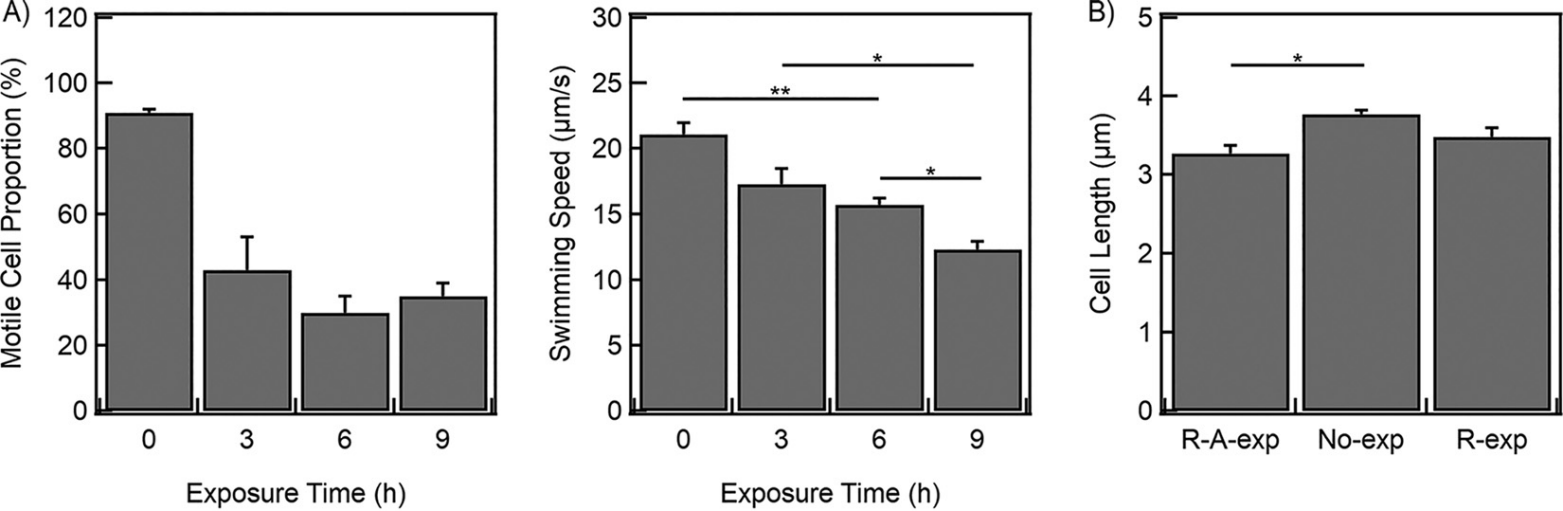
(A) Motile proportion and mean speed of swimming cells of the *R-A-exp* cells in the presence of ampicillin. The means and standard errors were calculated over three biological replicates. Motile proportions were determined from ~130 to 270 cells per replicate. Swimming speeds were determined from ~10 to 225 cells per replicate. (B) Cell length of *R-A-exp*, *No-exp*, and *R-exp* cells. The means and standard errors were calculated over three independent biological replicates with a total of 105 cells for each sample group. *, *P* < 0.05; **, *P* < 0.01.

To determine if motility was stable in the *R-A-exp* population over time, we drew samples every 3 h for a total duration of 9 h without removing ampicillin. As observed from the results in Fig. 2A, the motile proportion decreased to ~43% ± 10% within the first 3 h. The proportion dropped to ~30% ± 5% after 6 h and did not decrease thereafter. Thus, most of the motility was lost in the first 3 h of exposure to ampicillin. The mean speeds decreased linearly to ~57% of the value for untreated cells over the 9 h (Fig. 2A).

We quantified the lengths of cells in the *R-A-exp*, *No-exp*, and *R-exp* populations. The differences in means were not significant (*P* > 0.05) except between *R-A-exp* and *No-exp*, with the former appearing slightly shorter (Fig. 2B). We were unable to quantify cell lengths in the *A-exp* population because the antibiotic lysed most of the cells and they were not visible under the microscope (Fig. S1). Considering that the mean cell length in the *R-A-exp* population was not higher than that in the *No-exp* population, it is likely that the 30-min exposure to rifampicin inhibited growth in a fraction of the population, promoting tolerance of ampicillin during subsequent exposure to the antibiotic.

10.1128/mbio.02384-22.1 FIG S1 Representative images from each treatment group. Download FIG S1, TIF file, 2.5 MB. Copyright © 2023 Lee et al. 2023 Lee et al. https://creativecommons.org/licenses/by/4.0/ This content is distributed under the terms of the Creative Commons Attribution 4.0 International license.

We then tested how the order of sequential exposure impacted motility. Cells were first exposed to ampicillin for 30 min, followed by the removal of ampicillin. They were subsequently exposed to rifampicin for 3 h. Motility was completely lost at the end of the rifampicin exposure. Thus, the pretreatment with ampicillin did not appear to preserve motility in the presence of rifampicin.

Next, we performed experiments to determine whether cells could survive and retain motility in the presence of ampicillin if they were pretreated with a translational inhibitor, such as tetracycline (37). We exposed the cells to tetracycline for 30 min; the working concentration of tetracycline was optimized to obtain cells that subsequently adapted to ampicillin in a proportion similar to that in the *R-A-exp* cells (see Materials and Methods). After washing the treated cells, we resuspended them in ampicillin for 3 h (*T-A-exp* treatment). Approximately 0.4% ± 0.2% of the cells in the *T-A-exp* treatment survived after the 3-h exposure to ampicillin when compared to the survival of the *No-exp* cells (Fig. S2). Interestingly, only ~5.5% ± 1.1% of the cells were motile after 3 h, much less than the motile fraction in the *R-A-exp* cells (Fig. S2). This suggests that when cells were transcriptionally inhibited for a brief duration (30 min), a greater fraction of the motile population became tolerant of ampicillin than when cells were translationally inhibited.

10.1128/mbio.02384-22.2 FIG S2 Surviving proportion (left), motile proportion (middle), and mean speeds of swimming cells (right). The means and standard errors were calculated over three independent biological replicates (three technical replicates per biological sample). The differences in the mean CFU/mL counts were statistically significant across all the groups. The mean speeds were not significantly different between *No-exp* and *T-exp* cells (*P* > 0.05); the differences in the mean speeds across all the other groups were significant. Download FIG S2, TIF file, 2.7 MB. Copyright © 2023 Lee et al. 2023 Lee et al. https://creativecommons.org/licenses/by/4.0/ This content is distributed under the terms of the Creative Commons Attribution 4.0 International license.

The short-time exposure to rifampicin helped retain motility not only in the presence of ampicillin but also in the presence of ciprofloxacin, a fluoroquinolone that inhibits DNA replication (38) and promotes tolerance by inducing the SOS response (39–41). As discussed in the supplemental material (see Fig. S3), a large fraction (~65% ± 4%) of rifampicin-treated cells were motile after 3 h of exposure to ciprofloxacin.

10.1128/mbio.02384-22.3 FIG S3 Surviving proportion (left), motile proportion (middle), and mean speeds of swimming cells (right). The means and standard errors were calculated over three independent biological replicates (three technical replicates per biological sample). The differences in the mean CFU/mL counts were not statistically significant between *R-C-exp* and *R-exp* cells (*P* > 0.05); the differences in the mean CFU/mL counts across all the other groups were significant. The mean speeds were not significantly different across the different treatments. We performed separate experiments in which the rifampicin-treated cells were exposed to ciprofloxacin (*R-C-exp*). After 3 h of exposure to ciprofloxacin, ~0.09% ± 0.02% cells in the *R-C-exp* treatment survived when compared to the survival of the *No-exp* cells. The proportion of *R-C-exp* cells that retained motility (~65% ± 4%) was higher than that in the *R-A-exp* cells, and the mean speed of swimming *R-C-exp* cells (17.4 ± 1.4 μm/s) was similar to that of swimming *R-A-exp* cells. Interestingly, ciprofloxacin appeared to work more slowly than ampicillin, as a larger fraction of the population (~0.2%) survived 3 h of exposure to ciprofloxacin when the cells were not preexposed to rifampicin (*C-exp*), with the surviving fraction decreasing to ~0 over 6 h. Download FIG S3, TIF file, 2.7 MB. Copyright © 2023 Lee et al. 2023 Lee et al. https://creativecommons.org/licenses/by/4.0/ This content is distributed under the terms of the Creative Commons Attribution 4.0 International license.

### Some cells retain high membrane potential upon sequential exposure to antibiotics.

Considering that the motile *R-A-exp* cells swam at ~81% ± 5% of the *No-exp* swimming speeds even after 3 h of exposure to ampicillin, it is likely that those cells had a high PMF and/or membrane potential. However, adaptive or nonheritable resistance is characterized by low PMF. Hence, we measured the membrane potential in the *R-A-exp* population and compared it with that of the *No-exp* cells. We did this with a fluorescence microscopy assay detailed in our recent work (42). We employed a cationic reporter dye, thioflavin T (ThT), which is taken up by cells due to their negatively charged membranes (43); dissipation of the membrane charge leads to the release of the dye from the cell. When excited with an appropriate illumination wavelength, the strength of emission from the cells indicates the relative membrane potential.

**FIG 3:**
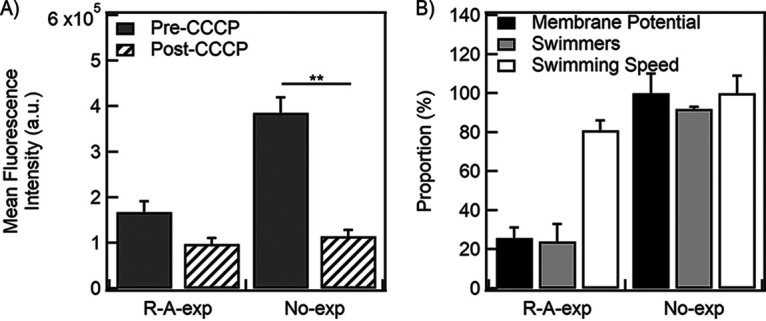
(A) The mean fluorescence intensities are indicated for the *R-A-exp* and *No-exp* cells before (dark-gray bars) and after (hatched bars) the addition of CCCP. Emissions were averaged across three biological replicates and were collected from ~500 to 1,000 cells per replicate. (B) The relative membrane potential calculated from equation 1 and the relative mean swimming speeds are indicated by the black and white bars, respectively. The percentages of swimming cells are indicated by the light-gray bars. The means and standard errors were calculated over three biological replicates. **, *P* < 0.01. a.u., arbitrary units.

We treated the *R-A-exp* cells with the dye and weakly illuminated them. The emissions were recorded with a highly sensitive photomultiplier, yielding the mean pre-CCCP signal level (*I*_pre_). Next, we fully dissipated the membrane potential with 25 μM CCCP and recorded the emissions again to obtain the mean post-CCCP signal level (*I*_post_). We also recorded the corresponding *I*_pre_ and *I*_post_ values for the *No-exp* control (Fig. 3A) and the *R-exp* treatment (Fig. S4). We calculated the relative mean membrane potential in the *R-A-exp* cells from equation 1 (28), as follows:

(1) $$\text{Membrane potential }\left(\text{\%}\right)=\frac{\Delta I_{\text{test}}}{\Delta I_{\text{control}}}\times 100\text{\%}$$

where $\Delta I=I_{\text{pre}}\;\;-\;\;\;I_{\text{post}}$.

10.1128/mbio.02384-22.4 FIG S4 (A) The mean fluorescence intensities are indicated for the *R-A-exp* treatment and controls: dark-gray bars indicate prestimulus values, and hatched bars indicate poststimulus values (following the addition of CCCP). Emissions were averaged across three biological replicates and were collected from ~500 to 1,000 cells per replicate. (B) The relative membrane potential calculated from equation 1 and the relative mean swimming speeds are indicated by the black and white bars, respectively. The percentages of swimming cells are indicated by the light-gray bars. The mean values and standard errors were calculated over three biological replicates. Download FIG S4, TIF file, 1.9 MB. Copyright © 2023 Lee et al. 2023 Lee et al. https://creativecommons.org/licenses/by/4.0/ This content is distributed under the terms of the Creative Commons Attribution 4.0 International license.

The mean membrane potential in the *R-A-exp* cells was ~26% ± 5% relative to the membrane potential of the *No-exp* cells (Fig. 3B). This was similar to the percentage of swimming cells in the *R-A-exp* population (~24% ± 9%). These measurements were consistent with the notion that the swimming fraction of the *R-A-exp* cells retained a high membrane potential, whereas the nonswimming population had a very low membrane potential, yielding a mean membrane potential of the population that was lower than that observed in the *No-exp* culture. This also suggests that the distribution of the values of the membrane potential/PMF in the *R-A-exp* population is probably bimodal. Such bimodality may not necessarily be visible in fluorescence intensity distributions, as the high-PMF cells in the *R-A-exp* population could limit their dye uptake because of high efflux.

### Motile cells retain membrane integrity.

Exposure to environmental stressors, including antibiotics, can yield cells with compromised inner membrane integrity (44). Interestingly, some of these cells may be capable of swimming with compromised membranes (45). To determine if membrane integrity was lost in the motile *R-A-exp* cells, we performed a standard live-dead assay. The assay involves a combination of two fluorescent dyes: propidium iodide (PI; red fluorescence), which enters the cytoplasm and labels nucleic acids only in cells with damaged membranes, and SYTO 9 (green fluorescence), which also labels nucleic acids (46). We stained the *No-exp* and *R-A-exp* cell populations with the two dyes and observed them by fluorescence microscopy. The untreated population (*No-exp*, *n* = 115 cells) had a high percentage of cells (~93% ± 2%) with intact membranes that appeared in the green channel. In comparison, ~68% ± 5% of the *R-A-exp* cells (*n* = 325 cells) were visible in the green channel. The rest were visible in the red channel, indicating that they had compromised membranes. The green channel showed several swimming *R-A-exp* cells and rotating/tethered cells, as well as nonmotile cells (Movie S1). The red channel showed only nonmotile cells; we failed to observe either swimmers or rotators (Movie S2). As no motile cells appeared in the red channel, the motile cells in the *R-A-exp* population did not have compromised membranes and were likely viable.

10.1128/mbio.02384-22.6 MOVIE S1 Motile and nonmotile cells from the *R-A-exp* population are visible in the green channel. Download Movie S1, AVI file, 8.3 MB. Copyright © 2023 Lee et al. 2023 Lee et al. https://creativecommons.org/licenses/by/4.0/ This content is distributed under the terms of the Creative Commons Attribution 4.0 International license.

10.1128/mbio.02384-22.7 MOVIE S2 Only nonmotile cells from the *R-A-exp* population are visible in the red channel. Download Movie S2, AVI file, 0.4 MB. Copyright © 2023 Lee et al. 2023 Lee et al. https://creativecommons.org/licenses/by/4.0/ This content is distributed under the terms of the Creative Commons Attribution 4.0 International license.

### Motile cells are viable and culturable.

Previous work reported that among the cells that develop adaptive resistance, not all cells retain the ability to reproduce when the antibiotic is removed; some cells are viable but cannot be cultured—termed viable but nonculturable cells (47). To determine if the motile cells in the *R-A-exp* population were able to divide when the antibiotic concentration was decreased, we performed single-cell growth assays. We washed the *R-A-exp* cells in tryptone broth (TB) after the 3-h exposure to ampicillin to remove the antibiotic. We then added the cell suspension to the top of an agarose pad and continuously recorded a preselected region of interest at the agarose surface. The motile cells quickly swim in and out of the region of interest, and hence, their growth cannot be tracked without immobilizing them. However, some swimmers will eventually approach the surface and adhere within the region of interest, as will nonmotile cells. The continuous recording creates a history of the cell phenotype prior to immobilization on the surface. We continued to record changes in cell lengths and division events over the next 5 h at 37°C. Each adherent cell within the region of interest was labeled nonmotile or motile by revisiting the initial part of the recorded video. Evaporation-induced changes in the gel surface caused different parts of the agar to go out of focus, making it challenging to visualize the cells over durations longer than a few hours.

**FIG 4:**
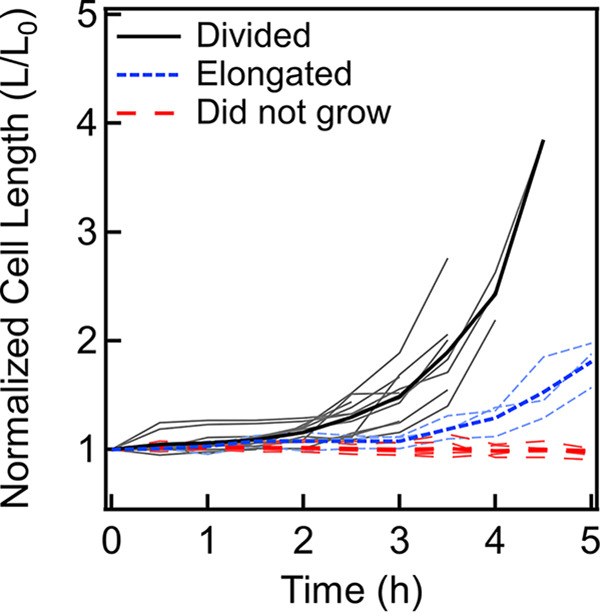
The rates of growth of different motile cells upon the removal of the antibiotic are plotted. The cell lengths (L) are normalized by their initial lengths (L_0_) at the beginning of observation. Light curves indicate values for individual cells, and bold curves indicate mean values. Three types of motile cells are indicated: elongating-dividing cells (black curves, *n* = 11), elongating cells (blue curves, *n* = 3), and nonelongating-nondividing cells (red curves, *n* = 8).

As shown by the results in Fig. 4, out of 22 motile *R-A-exp* cells, 11 elongated and divided (see also Fig. S5). Three cells elongated but did not divide within the duration of observation. The remaining 8 cells did not elongate or divide within the observation time. Thus, ~65% of the motile cells in the *R-A-exp* population were viable and culturable. Given the relatively short duration of observation, it was not possible to assess the ability of the remaining 35% of the motile cells to grow. Although we did not quantify growth in nonmotile cells, some of them were observed to grow and divide within this duration.

10.1128/mbio.02384-22.5 FIG S5 Changes in cell length of motile *R-A-exp* cells upon the removal of ampicillin. Three types of motile cells are indicated: elongating cells that divided during observation (black curves, *n* = 11 cells), elongating cells that did not divide during observation (blue curves, *n* = 3), and nonelongating-nondividing cells (red curves, *n* = 8). Download FIG S5, TIF file, 1.8 MB. Copyright © 2023 Lee et al. 2023 Lee et al. https://creativecommons.org/licenses/by/4.0/ This content is distributed under the terms of the Creative Commons Attribution 4.0 International license.

## DISCUSSION

In this study, we sequentially exposed cells to two antibiotics, rifampicin and ampicillin, and measured the motile behavior of surviving cells (*R-A-exp* population). In agreement with earlier works (17, 18), cells that were preexposed to rifampicin adapted to ampicillin without gaining any genetic mutations that could confer long-term resistance. This nonheritable resistance or tolerance was reminiscent of the persister phenomenon (15), although the *R-A-exp* cells most likely differed from the spontaneous persisters that emerge in cultures stochastically (48). Our key finding was that a significant fraction of the *R-A-exp* population swam rapidly for several hours in the presence of ampicillin. This suggested that a part of the *R-A-exp* population retained high PMF, as PMF dissipation inhibits motility in E. coli (28). As this fraction is relatively small (~30%), it is unlikely to be detected in population-based measurements—for example, in measurements of cellular respiration (18). The high-PMF cells remained viable during ampicillin exposure and were able to grow upon the removal of the antibiotic.

Measurements of membrane potential in the *R-A-exp* cells were consistent with the notion that the population consisted of a mix of at least two phenotypic states: the motile fraction containing cells with high PMF and a nonmotile fraction in which cells had dissipated their PMF. Presumably, the exposure to rifampicin inhibited cell wall synthesis in a small population of the cells, which helped them survive against lethal concentrations of ampicillin. In this small population, one fraction of cells survived without dissipating the PMF, and another survived by dissipating the PMF. It is likely that the two fractions employed different mechanisms to tolerate ampicillin. Mechanisms that can promote tolerance of antibiotics under stress include those that activate the SOS response pathway, which induces toxins like TisB or HokB and dissipates the PMF (41, 49). In E. coli, low PMF levels deplete ATP levels, as the PMF provides the driving force for ATP synthesis during respiratory growth (50). Low PMF and ATP levels correlate positively with cell dormancy, which promotes persistence (17, 51). Additionally, several other toxin-antitoxin pairs and high levels of (p)ppGpp can also induce cell dormancy (25, 41, 52–54). Such dormancy likely underpinned the tolerance of ampicillin in the low-PMF fraction of the *R-A-exp* population.

Cell dormancy, though, is unlikely to be necessary for adaptation to antibiotic stress (55, 56). It is possible that the high-PMF fraction survived with the aid of efflux pumps, which are powered by PMF. As cells that were not pretreated with rifampicin did not adapt to ampicillin (*A-exp*), the efflux activity in the high-PMF *R-A-exp* cells was probably elevated compared to the basal activity in cells that were never exposed to rifampicin. High efflux is effective in decreasing the intracellular concentrations of a variety of antibiotics (20, 57, 58), and it can inhibit growth (59–61). The elevated efflux and the inhibited growth likely helped the motile *R-A-exp* cells survive for several hours in the presence of ampicillin.

The motile *R-A-exp* cells did not swim faster than wild-type cells, which indicated that their PMF levels were not any higher than those of the wild-type cells. Thus, if enhanced efflux activity was indeed the cause of survival of the motile *R-A-exp* cells, the enhancement might have occurred through a transcriptional response to rifampicin. Indeed, rifampicin can transiently upregulate *rpoB* expression to promote tolerance (62–65). To test this idea, we inhibited translation by pretreating cells with tetracycline in place of rifampicin. Although the tetracycline-treated cells exhibited a tolerance of ampicillin (*T-A-exp* cells) similar to that of the *R-A-exp* cells, only ~5% of the *T-A-exp* cells were motile in the presence of ampicillin. We interpret the latter result to mean that most of the high-PMF cells were eliminated in the *T-A-exp* population, presumably because tetracycline prevented the synthesis of new efflux pumps.

Our results show that *R-A-exp* cells can swim in the presence of ampicillin for at least 9 h, which indicates that they retain PMF over long durations. The most significant drop in motility in the *R-A-exp* fraction occurred within the first 3 h of ampicillin exposure, indicating that ampicillin eliminated much of the population that failed to dissipate its PMF within this duration. In the presence of certain stressors, aminoglycosides, for example, cells that retain PMF may not survive since PMF helps internalize the compound (22). The cost of retaining PMF is likely balanced by the benefit conferred by motility, as the latter could help cells swim out of deleterious environments. Flagellar rotation speeds being proportional to the PMF, it is possible to quantify the PMF from swimming speeds (27, 28). Considering that the swimming speeds decreased gradually, there must have been a deficit in PMF generation compared to its utilization. Once free from the antibiotic, the motile cells did not resume growth immediately. However, approximately 2 h later, growth began, followed by cell division in most cases. Some cells took longer to resuscitate; it is possible that given adequate observation time, they would have divided too.

With the growing threat of antibiotic resistance worldwide, sequential administration of antibiotics has received increasing attention as an effective strategy to eradicate bacterial pathogens. Evidently, the order of antibiotic exposure is important, as it influences the likelihood of nonheritable resistance. Also, the heterogeneity we have observed in the adapted populations—low- and high-PMF cells—is likely a part of bet-hedging strategies to enhance fitness. These results highlight the need to identify different survival mechanisms within clonal populations to improve the effectiveness of sequential therapies.

## MATERIALS AND METHODS

### Bacterial strains and growth conditions.

Experiments were performed with E. coli K-12 strains RP437 and AW405, as these have been widely used for motility studies (29, 66). Fresh colonies were streaked on lysogeny broth (LB) agar plates from frozen glycerol stocks for each experiment. Single colonies were inoculated into 25 mL of tryptone broth (TB) in 250-mL Erlenmeyer flasks and incubated at 33°C in a shaker set at 250 rpm for 16 h. Subsequently, each culture was diluted 1:1,000 in 25 mL fresh TB and grown to an OD_600_ of 0.8 at 33°C with shaking at 250 rpm.

### Selection of antibiotic concentrations.

The MICs were determined by measuring the inhibition of growth in freshly inoculated cultures in 2 mL TB in 12-well plates. For each antibiotic, the medium in the plate was supplemented with 10 different concentrations, with the remaining two wells containing the positive and negative controls. The plates were incubated for 16 h at 33°C with shaking at 250 rpm. The MIC was determined qualitatively by observing lack of turbidity from three biological replicates. Following earlier work (17), the working concentrations of antibiotics selected were several factors higher than the MIC values, as follows: rifampicin, 100 μg/mL (MIC = 16 μg/mL); ampicillin, 100 μg/mL (MIC = 8 μg/mL); ciprofloxacin, 25 μg/mL (MIC = 1.28 to 2.56 μg/mL); and tetracycline, 5 μg/mL (MIC = 0.125 to 0.25 μg/mL). For tetracycline, the working concentration was optimized to obtain similar percentages of surviving cells in the *T-A-exp* treatment as in the *R-A-exp* treatment.

### Antibiotic exposure experiments.

For the *R-A-exp* treatment, we adopted previously established protocols for carrying out sequential exposures to antibiotics (17). Cells were grown to an OD_600_ of 0.8 and then supplemented with rifampicin (100 μg/mL) for 30 min (33°C and 250 rpm). Next, the cell suspension was centrifuged (1,000 × *g* for 7 min) and the supernatant was discarded. The cell pellet was gently resuspended in TB supplemented with ampicillin (100 μg/mL). Cells were incubated in ampicillin for 3 h (33°C and 250 rpm). For prolonged antibiotic treatment, ampicillin exposure was extended to 9 h. For the *A-exp* treatment, cells were grown to an OD_600_ of 0.8 and then supplemented with ampicillin (100 μg/mL) for 3 h (33°C and 250 rpm). For prolonged antibiotic treatment, ampicillin exposure was extended to 9 h. Finally, for the *No-exp* treatment, cells were grown to an OD_600_ of 0.8 without any exposure to antibiotics.

### Viability assay.

At each time point, 0.1-mL volumes were drawn from the cell suspension, serially diluted in phosphate-buffered saline (PBS), and plated on LB agar plates. Colonies were counted after 16 h of incubation at 37°C to determine the number of surviving cells. To determine whether any spontaneous resistant mutants had arisen during the growth of the cultures, cells were also plated on LB agar plates supplemented with ampicillin. No spontaneous resistance was observed.

### Motility assay.

At each time point, 0.1-mL volumes were drawn from the cell suspension and diluted in TB. The diluted suspensions were introduced to tunnel slides prepared by sticking two glass surfaces together with double-sided adhesive tape (67). We used a Nikon Optiphot microscope equipped with a 20× phase objective to visualize cell motility. A charge-coupled-device (CCD) camera (UI-3240LE; IDS Imaging) was employed to record videos at 45 frames per second.

### Motility and cell length analysis.

We determined the motile proportion and swimming speeds with custom-written particle-tracking algorithms (68). Briefly, our codes identified all the cells in each frame with a brightness-weighted centroid detection algorithm and linked them in subsequent frames (69). We time averaged the total number of cells in the field of view over all the frames to obtain the mean population of cells, *N*. To distinguish motile cells (*M*) from nonmotile cells (*I*), we first calculated the instantaneous displacements for each cell between consecutive frames; instantaneous speeds were calculated by multiplying these displacements by the frame rate. Different cells were observed in the camera’s field of view for different durations. To accurately calculate quantitative values (motile proportion and swimming speeds), we employed thresholds for the overall displacements and the speeds. According to our observations, nonmotile cells exhibited instantaneous speeds that could be as high as ~3 to 4 μm/s due to hydrodynamic drift. Hence, a cell was considered motile if its speed was >6 μm/s. Nonmotile cells sometimes transiently appeared and disappeared from the field of view because of diffusion or drift, giving the false impression of cells swimming at very high speeds. Hence, we imposed an additional requirement for motility: a cell had to cover a minimum distance of ~2 μm in 1/3 of a second without disappearing from the field of view in that duration. In addition to swimmers, we found that motile cells occasionally adhered to the glass surface such that they would rotate similarly to tethered cells (33). These cells were detected by the algorithm separately and included in the motile population (*M*). The motile proportion was finally calculated from the ratio of *M* and *N*; the swimming proportion was calculated by subtracting the surface-adhered rotating cells from the motile population and dividing the remainder by *N*. Swimming speeds were determined from the swimming proportion only.

The cell lengths were quantitatively determined from individual images by fitting an ellipse on each cell with a freely downloadable MATLAB code (70). The code outputs the major axis (length) of the fitted ellipse.

### Membrane potential measurements.

We used the dye thioflavin T (ThT) to measure the membrane potential, following earlier work (28). Following treatment with rifampicin and ampicillin (*R-A-exp* cells) or no treatment (*No-exp* cells), the 10-mL cell suspensions were pelleted by centrifugation (1,500 × *g* for 5 min) and resuspended in 10 mL motility buffer (MB) (0.01 M potassium phosphate, 0.067 M NaCl, 0.1 mM EDTA, 1 μM methionine, 10 mM lactic acid, pH 7.0). They were pelleted again and resuspended in 10 mL MB supplemented with 10 μM ThT (MB-ThT solution). They were pelleted for the last time and concentrated 10-fold by resuspension in 1 mL MB-ThT solution. Volumes of 50 μL of cell suspensions were placed on 12-mm-diameter coverslips that had been treated with 0.01% poly-l-lysine for 5 min and then washed in copious amounts of MB. The coverslips were placed in a perfusion chamber that allowed a constant flow of MB-ThT solution from a reservoir (28, 42). A Nikon Ti-E microscope with a 60× water immersion objective was used to image the cells. Coverslips were scanned to select regions with maximum and uniform cell coverage. At any given time, there were ~500 to 1,000 cells in the field of view.

The cells were excited with a light-emitting diode (LED) illumination source (SOLA SE II 365 Light Engine; Lumencor) filtered with a 435-/20-nm excitation filter (Nikon). The emissions were passed through a 525-/50-nm emission filter (AVR Optics) and relayed to a sensitive photomultiplier tube (H7421-40 SEL; Hamamatsu Corporation). The photon counts were recorded with a custom-written LabVIEW code at a sample rate of 10 Hz. In each experiment, emissions were recorded from four different regions on the coverslip while noting the exact *x*-*y* coordinates on the automated stage (MS-2000; Applied Scientific Instrumentation). For each region, emissions were recorded for ~150 s and a mean value was calculated. After the emissions were recorded, the perfusing reservoir was switched to one containing MB-ThT solution supplemented with 25 μM carbonyl cyanide *m*-chlorophenyl hydrazine (CCCP). The four regions on the coverslip were revisited, and emissions were recorded postexposure to CCCP. The emissions recorded after CCCP exposure provided the background fluorescence signal, as CCCP dissipates the membrane potential.

### Live-dead assay.

The LIVE/DEAD BacLight bacterial viability kit (L7012; Invitrogen) was used to evaluate membrane integrity following the manufacturer’s instruction. A mixture of two fluorescent dyes, SYTO 9 and propidium iodide (PI), was used. Following treatment with rifampicin and ampicillin (*R-A-exp* cells) or no treatment (*No-exp* cells), the 10-mL cell suspensions were pelleted by centrifugation (1,000 × *g* for 7 min) and resuspended in 10 mL MB. They were pelleted again and resuspended in MB to achieve an OD_600_ of 0.2. Cell suspensions were labeled with both dyes, SYTO 9 (1.67 μM) and PI (20 μM), and vortexed at 500 rpm for 15 min at room temperature. The cells were imaged on a Nikon Ti-E microscope with a 20× objective. A scientific complementary metal oxide semiconductor (sCMOS) camera (Andor Zyla; Oxford Instruments) was used to record videos at 0.05 frames per second.

### Motility agarose pad assay.

We prepared 1% agarose pads by melting low-melting-point agarose in LB medium. We poured a thin layer of the agarose-LB medium on a culture dish (Delta T Culture Dish; Bioptechs). After drying the agarose pad, a dilute suspension of prewashed 2-μm beads was added to the top to help locate the agar surface. We then washed 3 mL of *R-A-exp* cells by centrifugation (1,000 × g for 7 min), followed by gentle resuspension in an equal volume of TB to avoid shearing the flagella. The cells were pelleted again and diluted 5-fold by resuspension in 15 mL TB. The diluted cell suspension was added to the pad surface. We imaged the cells immediately using a Nikon Optiphot microscope equipped with a 20× phase objective. The microscope was kept inside an environmental chamber (M 5506; Electro-Tech Systems) that enabled the temperature and humidity to be maintained at 37°C and 70%, respectively. We monitored the agarose pad surface while recording videos with a CCD camera (UI-3240LE; IDS Imaging) at 20 frames per second. Over time, both motile and nonmotile cells adhered to the surface. Once the cells adhered, video recording was stopped. Thereafter, images were taken every 30 min for 5 h. During analysis, we revisited the recorded videos to distinguish the motile cells from the nonmotile ones just prior to adhesion.

### Statistical analysis.

All statistical analyses were performed with Student’s *t* test, with equal or unequal variances as appropriate. Results with a *P* value of <0.05 were considered statistically significant and have been indicated in figures with asterisks. For small sample sizes, a one-sided Wilcoxon-Mann-Whitney test was used.

### Data availability.

All data are included in the manuscript and supplemental material. Strains are available for purchase from the Coli Genetic Stock Center.
