# Supplementary figures and images for: Heterogeneous Distribution of Proton Motive Force in Nonheritable Antibiotic Resistance
Source: mBio. 2023 Jan 4;14(1):e02384-22. doi: 10.1128/mbio.02384-22 (PMC9973297; doi:10.1128/mbio.02384-22)

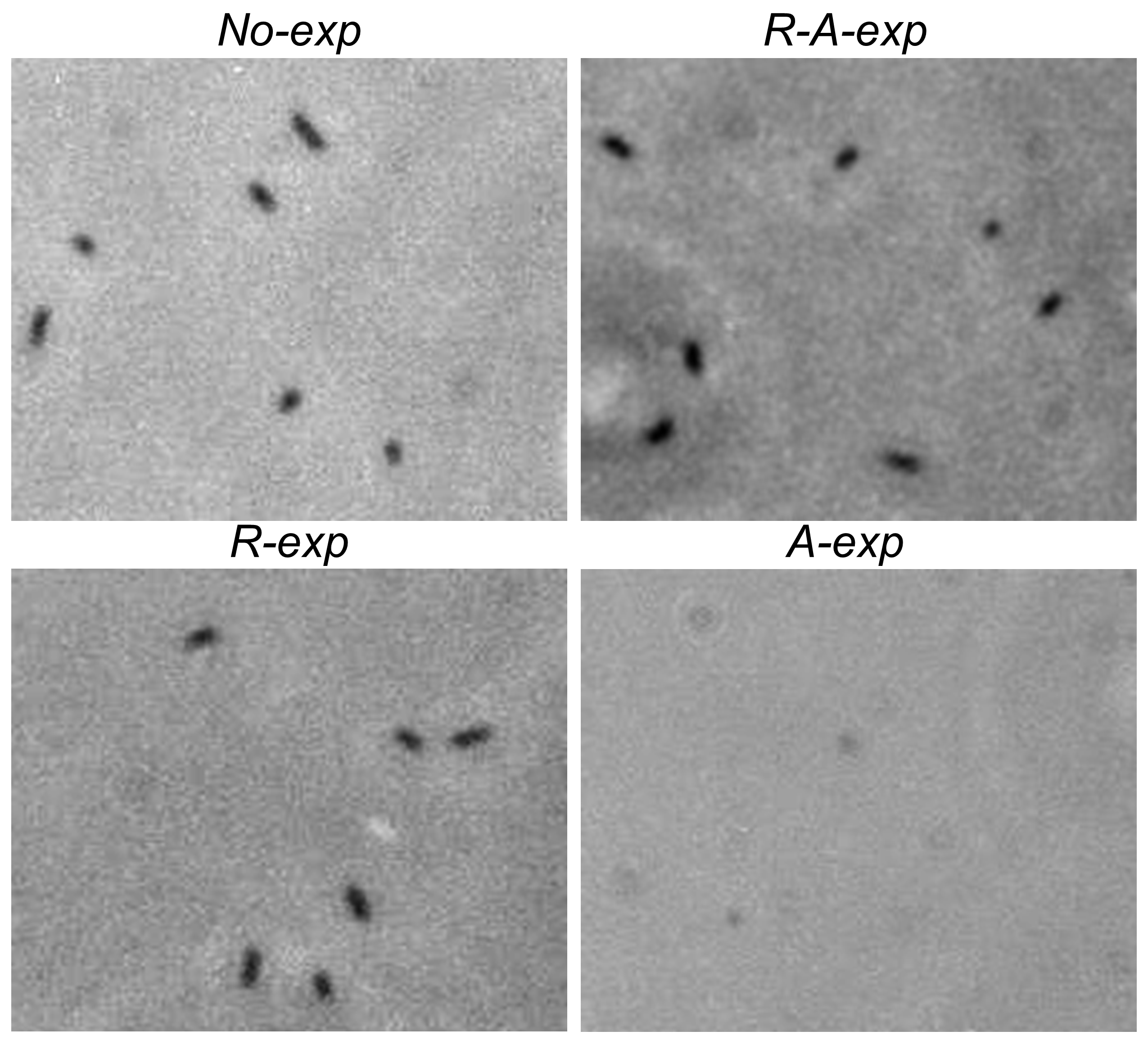

Supplement: FIG S1 [file mbio.02384-22-s0001.tif]

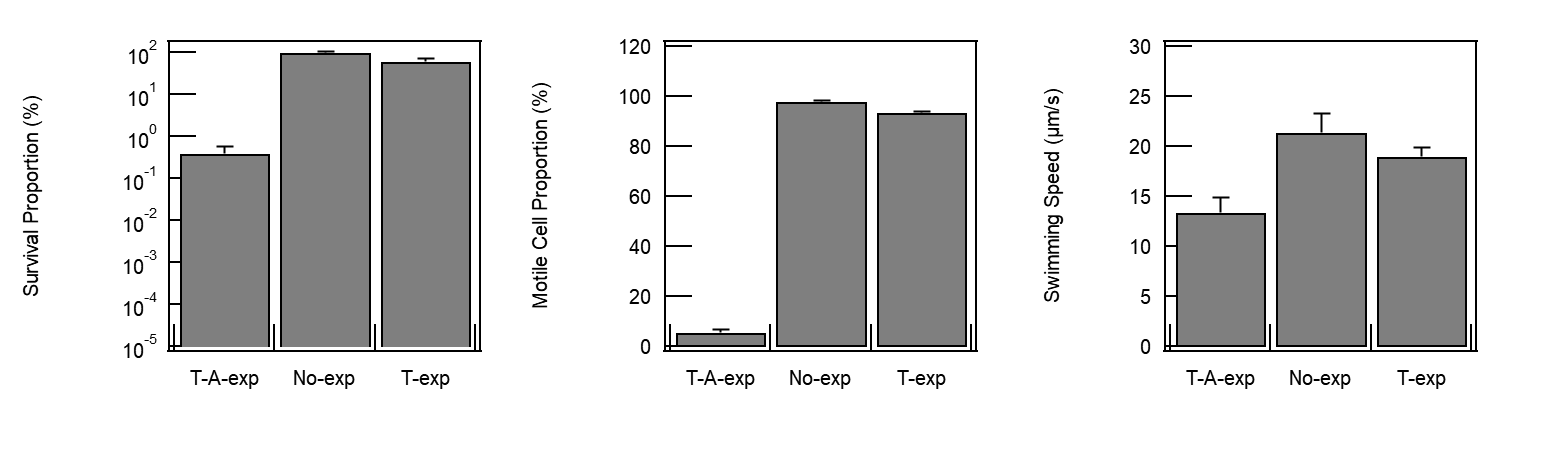

Supplement: FIG S2 [file mbio.02384-22-s0002.tif]

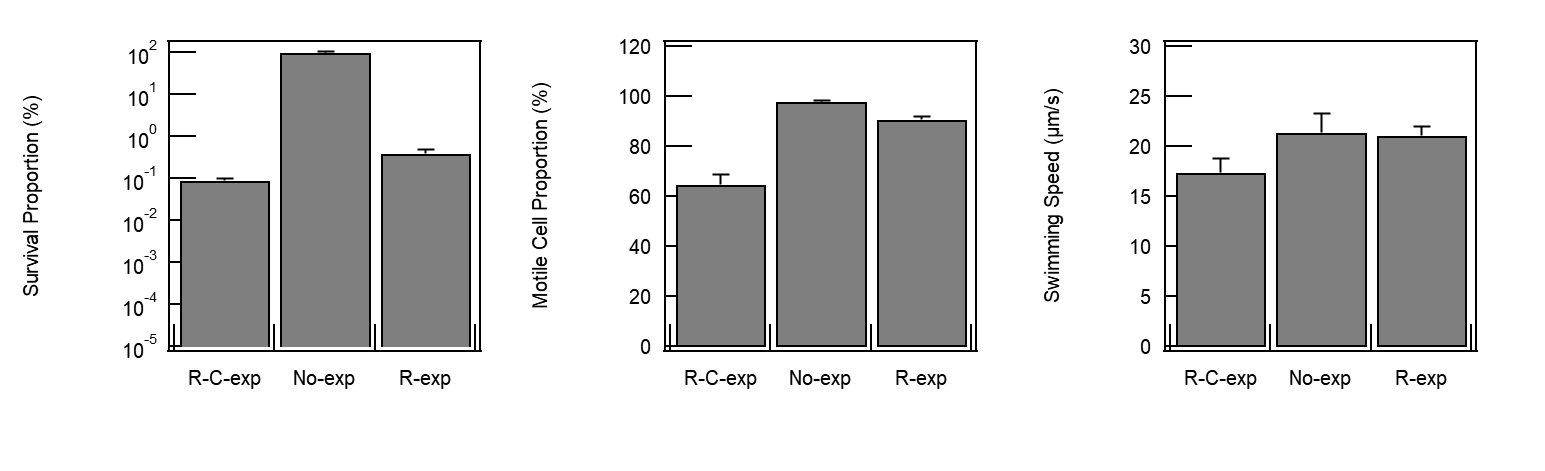

Supplement: FIG S3 [file mbio.02384-22-s0003.tif]

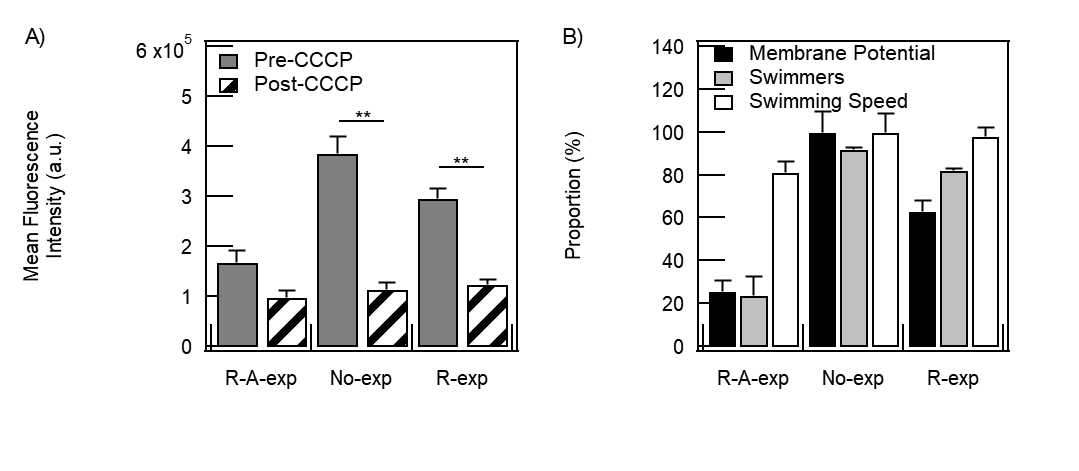

Supplement: FIG S4 [file mbio.02384-22-s0004.tif]

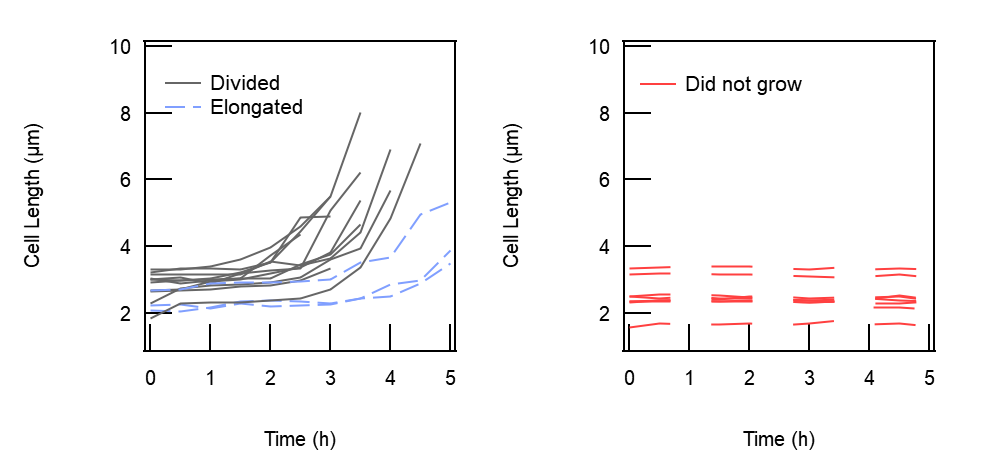

Supplement: FIG S5 [file mbio.02384-22-s0005.tif]
